# COVID-19 Risk Factors and Mortality Outcomes Among Medicare Patients Receiving Long-term Dialysis

**DOI:** 10.1001/jamanetworkopen.2021.35379

**Published:** 2021-11-17

**Authors:** Stephen Salerno, Joseph M. Messana, Garrett W. Gremel, Claudia Dahlerus, Richard A. Hirth, Peisong Han, Jonathan H. Segal, Tao Xu, Dan Shaffer, Amy Jiao, Jeremiah Simon, Lan Tong, Karen Wisniewski, Tammie Nahra, Robin Padilla, Kathryn Sleeman, Tempie Shearon, Sandra Callard, Alexander Yaldo, Lisa Borowicz, Wilfred Agbenyikey, Golden M. Horton, Jesse Roach, Yi Li

**Affiliations:** 1 University of Michigan, Kidney Epidemiology and Cost Center, Ann Arbor; 2 Department of Biostatistics, University of Michigan, Ann Arbor; 3 Division of Nephrology, University of Michigan Health System, Ann Arbor; 4 Department of Health Policy and Management, University of Michigan, Ann Arbor; 5 Centers for Medicare & Medicaid Services, Baltimore, Maryland

## Abstract

**Importance:**

There is a need for studies to evaluate the risk factors for COVID-19 and mortality among the entire Medicare long-term dialysis population using Medicare claims data.

**Objective:**

To identify risk factors associated with COVID-19 and mortality in Medicare patients undergoing long-term dialysis.

**Design, Setting, and Participants:**

This retrospective, claims-based cohort study compared mortality trends of patients receiving long-term dialysis in 2020 with previous years (2013-2019) and fit Cox regression models to identify risk factors for contracting COVID-19 and postdiagnosis mortality. The cohort included the national population of Medicare patients receiving long-term dialysis in 2020, derived from clinical and administrative databases. COVID-19 was identified through Medicare claims sources. Data were analyzed on May 17, 2021.

**Main Outcomes and Measures:**

The 2 main outcomes were COVID-19 and all-cause mortality. Associations of claims-based risk factors with COVID-19 and mortality were investigated prediagnosis and postdiagnosis.

**Results:**

Among a total of 498 169 Medicare patients undergoing dialysis (median [IQR] age, 66 [56-74] years; 215 935 [43.1%] women and 283 227 [56.9%] men), 60 090 (12.1%) had COVID-19, among whom 15 612 patients (26.0%) died. COVID-19 rates were significantly higher among Black (21 787 of 165 830 patients [13.1%]) and Hispanic (13 530 of 86 871 patients [15.6%]) patients compared with non-Black patients (38 303 of 332 339 [11.5%]), as well as patients with short (ie, 1-89 days; 7738 of 55 184 patients [14.0%]) and extended (ie, ≥90 days; 10 737 of 30 196 patients [35.6%]) nursing home stays in the prior year. Adjusting for all other risk factors, residing in a nursing home 1 to 89 days in the prior year was associated with a higher hazard for COVID-19 (hazard ratio [HR] vs 0 days, 1.60; 95% CI 1.56-1.65) and for postdiagnosis mortality (HR, 1.31; 95% CI, 1.25-1.37), as was residing in a nursing home for an extended stay (COVID-19: HR, 4.48; 95% CI, 4.37-4.59; mortality: HR, 1.12; 95% CI, 1.07-1.16). Black race (HR vs non-Black: HR, 1.25; 95% CI, 1.23-1.28) and Hispanic ethnicity (HR vs non-Hispanic: HR, 1.68; 95% CI, 1.64-1.72) were associated with significantly higher hazards of COVID-19. Although home dialysis was associated with lower COVID-19 rates (HR, 0.77; 95% CI, 0.75-0.80), it was associated with higher mortality (HR, 1.18; 95% CI, 1.11-1.25).

**Conclusions and Relevance:**

These results shed light on COVID-19 risk factors and outcomes among Medicare patients receiving long-term chronic dialysis and could inform policy decisions to mitigate the significant extra burden of COVID-19 and death in this population.

## Key Points

**Question:**

What are the characteristics and mortality outcomes associated with COVID-19 among Medicare patients undergoing long-term dialysis?

**Findings:**

This cohort study among 498 169 patients receiving regular maintenance dialysis found several risk factors for COVID-19 that persisted as risk factors for mortality: nursing home status, time on dialysis, congestive heart failure, diabetes, and comorbidity burden. Higher COVID-19 rates were observed among Black patients, while attenuated survival differences were observed between Black and non-Black patients, and although male sex was not associated with a higher COVID-19 rate, it was associated with higher mortality among patients with COVID-19.

**Meaning:**

These findings suggest that among patients undergoing long-term dialysis, Black race, male sex, nursing home status, and having comorbidities, such as diabetes and cardiac diseases, were associated with higher risk of COVID-19 and higher post–COVID-19 mortality.

## Teaser

This cohort study examines risk factors associated with COVID-19 and mortality among Medicare patients receiving long-term maintenance dialysis.

## Introduction

In the United States, more than 600 000 COVID-19 deaths have been reported. Individuals with preexisting conditions, such as obesity, diabetes, or chronic cardiovascular, lung, liver, and kidney diseases, are at an elevated risk for adverse COVID-19 outcomes.^1,2,3,4,5,6^ There is also evidence of sex, race, and regional disparities for individuals with COVID-19 in the US.^7,8,9^

Patients with end-stage kidney disease (ESKD) are at a higher risk of worsened prognoses with COVID-19, especially those with additional comorbidities.^10,11,12,13,14,15,16,17,18,19,20,21^ Patients with ESKD undergoing maintenance hemodialysis are particularly susceptible to SARS-CoV-2 infection, as treatment necessitates frequent visits to outpatient dialysis units.^22,23,24,25,26,27^ As patients receiving long-term dialysis comprise 70% of the ESKD population,^28^ understanding their SARS-CoV-2 infection and mortality risk has sparked much interest. For example, recent work has modeled excess deaths during the first wave of the pandemic, adjusting for patient age and ESRD Network.^29^ Various studies have investigated the impact of COVID-19 on dialysis patients regionally or within specific dialysis organizations^21,30,31,32,33,34^; however, there is a lack of literature on this patient population nationally.

Leveraging a comprehensive national database, we examined the risk factors for COVID-19 and subsequent mortality among Medicare patients receiving long-term dialysis. For all included patients, we first identified which demographic and clinical features were associated with COVID-19. Then, among those with COVID-19, we examined the risk factors for mortality after COVID-19 diagnosis. We complemented our analysis with a third model on mortality prior to diagnosis, to gain a comprehensive understanding of mortality among patients receiving dialysis in the era of COVID-19.

## Methods

This cohort study is based on contractual work performed for the Centers for Medicare & Medicaid Services (CMS) to support kidney disease quality measure development and maintenance; therefore, it is exempt from institutional review board review and waived from Health Insurance Portability and Accountability Act of 1996 requirements for informed consent, per federal policy. This was a retrospective, claims-based analysis following the Strengthening the Reporting of Observational Studies in Epidemiology (STROBE) reporting guideline.

### Study Population

Our study population consisted of all Medicare patients receiving long-term dialysis in 2020, derived from the CMS clinical and administrative databases. Patients were considered on Medicare if they were enrolled in Medicare Advantage, had at least $1200 of Medicare-paid dialysis claims, or had at least 1 Medicare inpatient claim in a given month or either of the 2 prior months. Patients were followed from January 1, 2020, or the first date in 2020 when they were Medicare-eligible and had been receiving regular maintenance dialysis for at least 90 days, whichever came later.

### National Mortality Trends

We compared mortality trends in 2020 with historic trends in 2013 to 2019 by deriving monthly empirical hazards for all-cause mortality: number of deaths / number at risk. Being at risk was defined as being a Medicare patient, assigned to a facility, and receiving dialysis; for a patient death to be attributed to the mortality hazards, the patient must have been at risk on the day they died. Results were calculated for all patients and stratified by race, sex, and locale.

### Identification of COVID-19 Diagnosis

We identified COVID-19 diagnoses from inpatient, outpatient, skilled nursing, home health, hospice, and physician/supplier claims; ESRD Death Notification forms (CMS Form 2746); and ESRD Medical Evidence forms for Medicare entitlement or new patient registration (CMS Form 2728). COVID-19 was identified in any claim carrying an *International Statistical Classification of Diseases and Related Health Problems, Tenth Revision* (*ICD-10*) code (B9729 or U07.1), as a condition on a patient’s Form 2728, as the primary or a secondary cause of death on a patient’s Form 2746, or through string matching of *COVID-19* and any typographical variants (eg, *COVIC*) written on these forms. Data were extracted from the CMS integrated data repository on May 17, 2021.

### Risk Factors for COVID-19 and Mortality

We identified several patient demographic and clinical characteristics reported on the patient’s medical evidence form, including age, sex, race (Black vs non-Black), ethnicity (Hispanic vs non-Hispanic), body mass index (BMI; calculated as weight in kilograms divided by height in meters squared), years receiving dialysis (ESKD vintage), and 13 comorbidities at ESKD incidence. Race was defined from CMS form 2728, and we dichotomized race because we were interested in studying the experience of Black patients. The non-Black reference group consists of White, Asian, Native Hawaiian or other Pacific Islander, and American Indian or Alaska Native patients, or those who reported other race. Additional prevalent comorbidities were identified from Medicare inpatient claims data, using recommendations from a 2015 technical expert panel on comorbidity risk adjustment for a mortality measure.^35^ Ninety condition categories, modified from the 2018 Agency for Healthcare Research and Quality Clinical Classifications Software diagnosis category set, were used to categorize these comorbidities. Using inpatient claims from 2019, we determined the number of condition groups a patient had in the previous calendar year. We included this number as a risk factor, as well as an indicator for patients having less than 6 months of Medicare claims in 2019. Six months was chosen based on prior practice to balance the accuracy of comorbidity information and the completeness of data when developing the National Quality Forum-endorsed mortality measure for dialysis facilities (eAppendix in the Supplement).^36^

Further important risk factors included patient locale, nursing home status, dialysis treatment modality, and Medicare type. We defined patient locale as urban or rural based on their zip code and the Office of Management and Budget’s definition of core-based statistical areas. To prevent confounding and potential survivorship bias, nursing home status was defined based on prior stays. Specifically, using the Medicare Minimum Data set (MDS), we determined if a patient was in a nursing home (long-term care or skilled nursing facility) for 0, 1 to 89, or 90 or more days in the 365 days prior to their time at risk. Using treatment history, sourced from Medicare claims and the Consolidated Renal Operations in a Web-Enabled Network database, we identified periods when a patient received either in-center hemodialysis or a home therapy (eg, peritoneal dialysis, home hemodialysis). Lastly, we determined if a patient was enrolled in Medicare Advantage vs Fee-for-Service using the Medicare Enrollment Database. We treated dialysis modality and Medicare Advantage status as time varying in our analysis. Additional references for data sources can be found in the eAppendix in the Supplement.

### Statistical Analysis

Descriptive statistics are presented as median with IQR for continuous variables and number with percentage for categorical covariates. To study the risk factors associated with COVID-19 and mortality, we fit 3 Cox proportional hazards models. In model 1, we studied patients’ time to COVID-19 diagnosis, which could be censored by death or the end of follow up (December 31, 2020). Patients were followed from January 1, 2020, or 90 days after being Medicare eligible and receiving regular maintenance dialysis, whichever came later. To maintain Medicare eligibility, we treated kidney transplantation and loss of Medicare eligibility as censoring events. In model 2, we studied the risk factors impacting postdiagnosis survival among patients with COVID-19. Postdiagnosis survival was defined as the time from COVID-19 diagnosis to death, which could be censored by kidney transplantation, Medicare ineligibility, or the end of follow up. Model 3 examined risk factors for death prior to COVID-19 diagnosis, the competing event for diagnosis examined in model 1. We modeled time to death, which could be censored by COVID-19 diagnosis, kidney transplantation, Medicare ineligibility, or the end of follow up. An illustration of the models and a breakdown of patient outcomes are presented in eFigure 1 and eFigure 2 in the Supplement. We report adjusted hazard ratios (HRs) and 95% CIs for the risk factors defined previously. As only 7 patients had missing covariate data, we proceeded with a complete case analysis. A 2-sided *P* < .05 was deemed significant. All analysis was conducted in R statistical software version 3.6.0 (R Project for Statistical Computing). Data were analyzed on May 17, 2021.

## Results

Among a total of 498 169 Medicare patients undergoing dialysis (median [IQR] age, 66 [56-74] years; 215 935 [43.1%] women and 283 227 [56.9%] men), 60 090 (12.1%) received a COVID-19 during the study period (Table 1). Most patients lived in an urban area (467 673 patients [94%]).

**Table 1.** Descriptive Characteristics for Medicare-Eligible Patients With ESKD Receiving Long-term Dialysis in 2020

Characteristic	No. (%) (N = 498 169)		
	Overall (n = 498 169)	Any COVID-19 Diagnosis	
		No (n = 438 079)	Yes (n = 60 090)
Age, median (IQR), y	66 (56-74)	66 (56-74)	66 (56-74)
Sex			
Women	215 935 (43.1)	187 830 (42.9)	27 105 (45.1)
Men	283 227 (56.9)	250 245 (57.1)	32 982 (54.9)
Race			
Black	165 830 (33.3)	144 043 (32.9)	21 787 (36.3)
Non-Black^a^	332 339 (66.7)	294 036 (67.1)	38 303 (63.7)
Ethnicity			
Non-Hispanic	411 298 (82.6)	364 738 (83.3)	46 560 (77.5)
Hispanic	86 871 (17.4)	73 341 (16.7)	13 530 (22.5)
BMI categories			
18.5-24.9	124 166 (24.9)	110 284 (25.2)	13 882 (23.1)
<18.5	13 911 (2.8)	12 463 (2.9)	1448 (2.4)
25.0-29.9	135 969 (27.3)	119 980 (27.4)	15 989 (26.6)
≥30	224 123 (45.0)	195 352 (44.6)	28 771 (47.9)
Locale			
Rural	30 496 (6.1)	26 791 (6.1)	3705 (6.2)
Urban	467 673 (93.9)	411 288 (93.9)	56 385 (93.8)
Nursing home stays in previous 365 d, d			
0	412 789 (82.9)	371 174 (84.7)	41 615 (69.3)
1-89	55 184 (11.1)	47 446 (10.8)	7738 (12.9)
≥90	30 196 (6.1)	19 459 (4.4)	10 737 (17.9)
Any Medicare advantage	137 889 (27.7)	126 167 (28.8)	11 722 (19.5)
Any in-center hemodialysis	443 013 (88.9)	386 783 (88.3)	56 230 (93.6)
ESKD vintage, median (IQR), y	3.8 (1.9-6.9)	3.8 (1.9-6.8)	4.0 (2.0-7.1)
Atherosclerotic heart disease	57 170 (11.5)	50 030 (11.4)	7140 (11.9)
Other cardiac disease	79 340 (15.9)	69 579 (15.9)	9761 (16.2)
Congestive heart failure	120 120 (24.1)	103 981 (23.7)	16 139 (26.9)
Inability to ambulate	20 648 (4.1)	16 747 (3.8)	3901 (6.5)
Chronic obstructive pulmonary disease	33 042 (6.6)	28 805 (6.6)	4237 (7.1)
Inability to transfer	9772 (2.0)	7744 (1.8)	2028 (3.4)
Malignant neoplasm, cancer	26 987 (5.4)	24 284 (5.5)	2703 (4.5)
Diabetes	69 169 (13.9)	60 288 (13.8)	8881 (14.8)
Peripheral vascular disease	39 924 (8.0)	34 576 (7.9)	5348 (8.9)
Cerebrovascular disease (CVA, TIA)	37 644 (7.6)	32 005 (7.3)	5639 (9.4)
Tobacco use (current smoker)	29 812 (6.0)	26 933 (6.1)	2879 (4.8)
Alcohol dependence	5736 (1.2)	5065 (1.2)	671 (1.1)
Drug dependence	5289 (1.1)	4638 (1.1)	651 (1.1)
No. of prevalent comorbidities	0.00 (0.00-4.00)	0.00 (0.00-4.00)	1.00 (0.00-5.00)

Abbreviations: BMI, body mass index (calculated as weight in kilograms divided by height in meters squared); CVA, cerebrovascular accident; ESKD, end-stage kidney disease; TIA, transient ischemic attack.

^a^ Race was dichotomized to focus on the experience of Black patients. The non-Black reference group included White, Asian, Native Hawaiian or other Pacific Islander, and American Indian or Alaska Native patients and those who reported other race.

### Risk Factors for COVID-19

COVID-19 rates were higher among Black (21 787 of 165 830 Black patients [13.1%] vs 38 303 of 332 339 non-Black patients [11.5%]) and Hispanic (13 530 of 86 871 Hispanic [15.6%] vs 46 560 of 411 298 non-Hispanic patients [11.3%]) patients. The most prominent differences in COVID-19 rates were observed between patients with short (ie, 1-89 days) nursing home stays (7738 of 55 184 patients [14.0%]) and extended (ie, ≥90 days) nursing home stays (10 737 of 30 196 patients [35.6%]) compared with patients who did not receive nursing home care in the prior year (41 615 of 412 789 patients [10.1%]).

Adjusting for all other risk factors and compared with no nursing home stay, prior short-term nursing home residence was associated with a 60% higher hazard for COVID-19 (HR, 1.60; 95% CI, 1.56-1.65), while extended stays were associated with a 448% higher hazard (HR, 4.48; 95% CI, 4.37-4.59). Black race (HR vs non-Black race, 1.25; 95% CI, 1.23-1.28) and Hispanic ethnicity (HR vs non-Hispanic ethnicity, 1.68; 95% CI 1.64-1.72), in addition to older age, higher BMI, congestive heart failure, inability to ambulate, diabetes, cerebrovascular disease, and higher prevalent comorbidity burden, were also associated with higher COVID-19 hazard. In contrast, urban residence, Medicare Advantage coverage, ESKD vintage, cancer, and tobacco use were associated with lower hazard of COVID-19 (Table 2).

**Table 2.** Risk Factors for COVID-19 Among Medicare-Eligible Patients With ESKD Receiving Long-term Dialysis in 2020

Characteristic	HR (95% CI)
Age (centered), per 10 y	1.02 (1.01-1.03)^a^
Sex	
Women	1 [Reference]
Men	1.01 (0.99-1.03)
Race	
Black	1.25 (1.23-1.28)^a^
Non-Black^b^	1 [Reference]
Ethnicity	
Non-Hispanic	1 [Reference]
Hispanic	1.68 (1.64-1.72)^a^
BMI categories	
18.5-24.9	1 [Reference]
<18.5	0.96 (0.91-1.01)
25.0-29.9	1.04 (1.02-1.07)^a^
≥30	1.09 (1.07-1.12)^a^
Locale	
Rural	1 [Reference]
Urban	0.96 (0.93-1.00)^a^
Nursing home stays in previous 365 d, d	
0	1 [Reference]
1-89	1.60 (1.56-1.65)^a^
≥90	4.48 (4.37-4.59)^a^
Modality	
In-center hemodialysis	1 [Reference]
Home dialysis	0.77 (0.75-0.80)^a^
Medicare Advantage	0.58 (0.57-0.59)^a^
ESKD vintage (centered), per 10 y	0.92 (0.90-0.94)^a^
Atherosclerotic heart disease	1.01 (0.99-1.04)
Other cardiac disease	1.01 (0.99-1.03)
Congestive heart failure	1.08 (1.06-1.10)^a^
Inability to ambulate	1.09 (1.04-1.14)^a^
Chronic obstructive pulmonary disease	1.03 (1.00-1.07)
Inability to transfer	1.00 (0.94-1.06)
Malignant neoplasm, cancer	0.91 (0.88-0.95)^a^
Diabetes	1.03 (1.01-1.06)^a^
Peripheral vascular disease	1.02 (0.99-1.05)
Cerebrovascular disease (CVA or TIA)	1.08 (1.05-1.11)
Tobacco use (current smoker)	0.84 (0.81-0.87)^a^
Alcohol dependence	0.97 (0.89-1.05)
Drug dependence	1.02 (0.94-1.11)
Prevalent comorbidities, per 1-unit increase	1.03 (1.03-1.03)^a^
<180 d of Medicare claims in 2019	1.43 (1.40, 1.47)^a^

Abbreviations: BMI, body mass index (calculated as weight in kilograms divided by height in meters squared); CVA, cerebrovascular accident; ESKD, end-stage kidney disease; HR, hazard ratio; TIA, transient ischemic attack.

^a^ Indicates statistical significance.

^b^ Race was dichotomized to focus on the experience of Black patients. The non-Black reference group included White, Asian, Native Hawaiian or other Pacific Islander, and American Indian or Alaska Native patients and those who reported other race.

### National Mortality Trends

**Figure 1.**
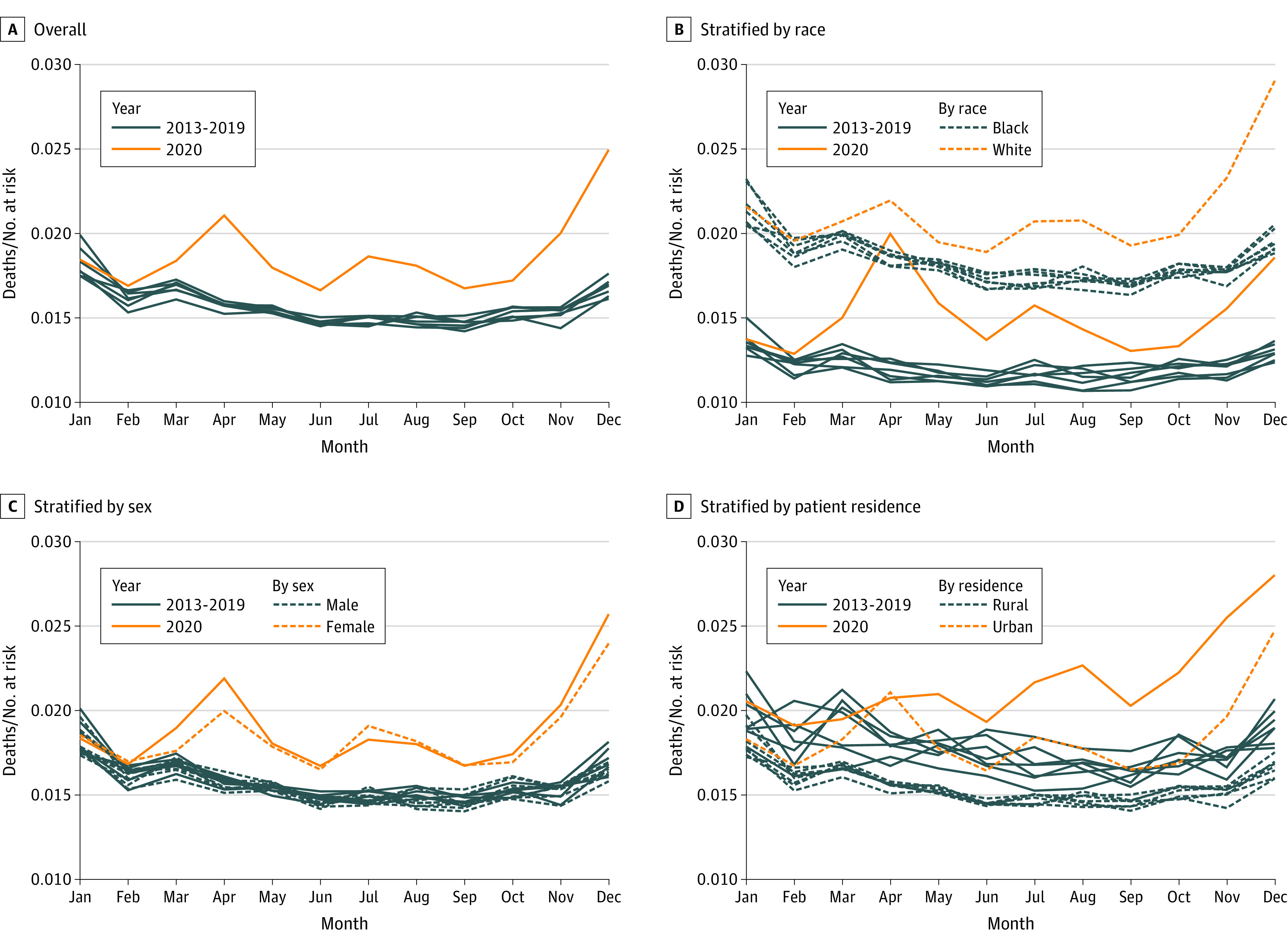
Monthly Empirical All-Cause Mortality Hazards Among Medicare Patients Receiving Dialysis From 2013 to 2020

Figure 1 compares monthly all-cause mortality hazard in 2020 with historical trends going back to 2013. Annual trends in 2013 to 2019 display a well-known seasonal pattern, with peaks in late January and early February.^37,38,39,40^ Beginning in 2020, deviations from this trend were commensurate with known waves of the COVID-19 pandemic (Figure 1A). Historically, Black patients receiving dialysis have had significantly lower mortality than non-Black patients in the US. However, in March through July 2020, Black patients had only lower mortality than non-Black patients (Figure 1B), with a narrower gap than prior years. Mortality for Black patients declined in May, returning close to normal historic differences by late summer. Sex differences were shown to be variable, although elevated hazards persisted throughout 2020 (Figure 1C). While mortality has been high since March 2020, the initial increase was markedly higher in urban areas. The spike in April was driven by a few key hotspots, namely New York, New York, and to a lesser extent, Detroit, Michigan, and Chicago, Illinois. These trends shifted as COVID-19 became more widespread (Figure 1D).

### Risk Factors for Postdiagnosis Mortality

**Figure 2.**
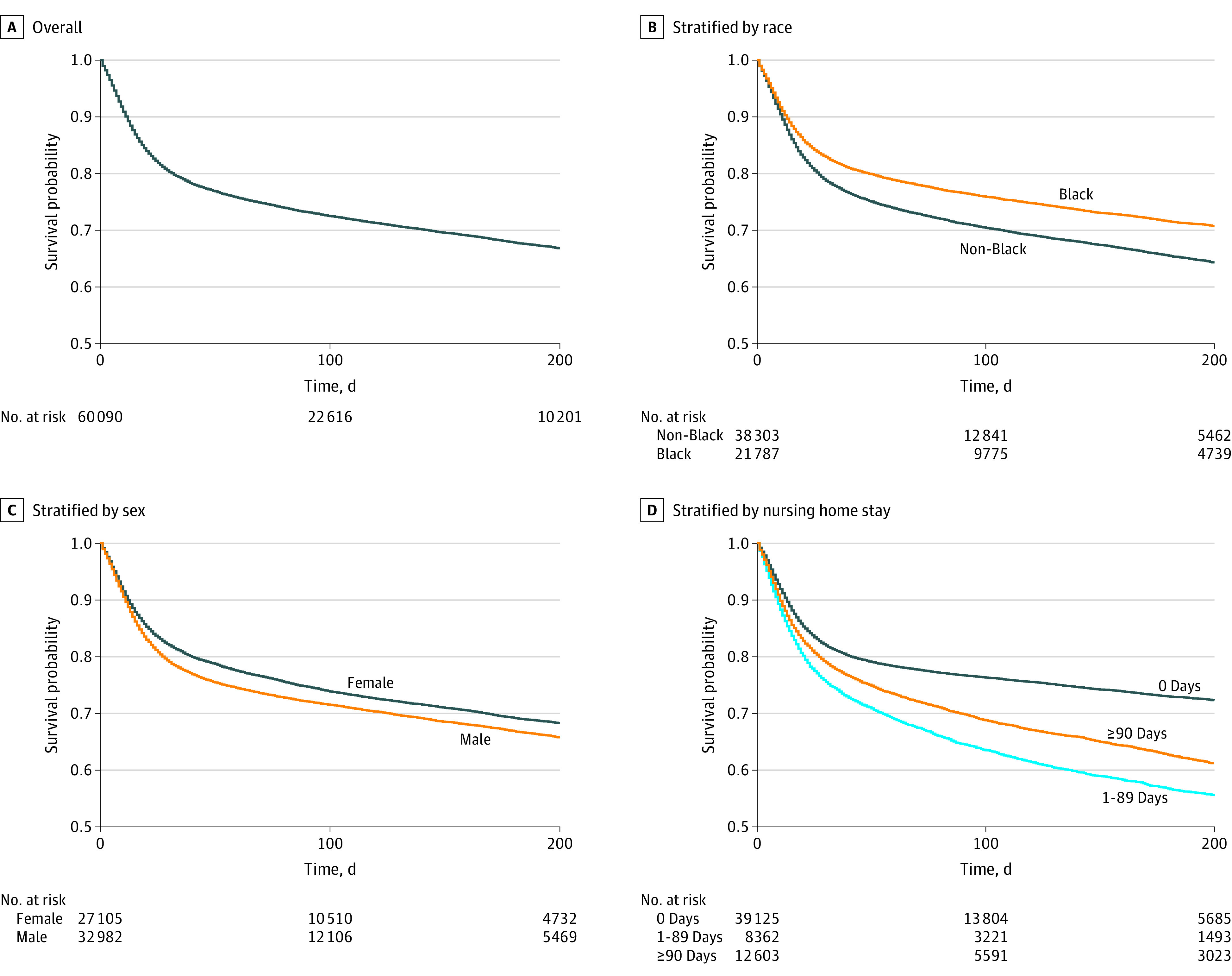
Kaplan-Meier Curves for All-Cause Mortality Among Medicare Dialysis Patients Diagnosed With COVID-19 Time at risk was taken from the date of a patient’s first Medicare claim carrying a COVID-19 diagnosis until kidney transplantation, death, or administrative censoring on December 31, 2020.

We observed 15 612 deaths (26.0%) among 60 090 patients with COVID-19, vs 72 339 deaths (16.9%) among 438 079 patients without COVID-19, indicating COVID-19 was associated with higher mortality in this population. Kaplan-Meier curves for post–COVID-19 survival are presented in Figure 2 and show attenuated differences in survival between Black and non-Black patients and worse survival outcomes among men and among patients with prior nursing home stays.

Age (centered HR per 10 years, 1.37; 95% CI, 1.35-1.39) and Hispanic ethnicity (HR vs non-Hispanic, 1.06; 95% CI, 1.02-1.10), in addition to higher BMI, congestive heart failure, and number of prevalent comorbidities were also associated with higher mortality after COVID-19. Residing in a nursing home for 1 to 89 days prior was associated with a 31% higher hazard for mortality (HR vs 0 days, 1.31; 95% CI, 1.25-1.37), while extended stays were associated with a 12% higher hazard for mortality (HR vs 0 days, 1.12; 95% CI, 1.07-1.16). Men had a 20% higher hazard of postdiagnosis mortality than women (HR, 1.20; 95% CI, 1.16-1.24). Black patients with COVID-19 had lower mortality hazard than non-Black patients (HR, 0.87; 95% CI, 0.84-0.90). Home dialysis (HR, 1.18; 95% CI, 1.11-1.25), in addition to longer ESKD vintage, Medicare Advantage coverage, and tobacco use, were associated with higher postdiagnosis mortality. Urban residence was associated with lower hazard for postdiagnosis mortality (HR vs rural, 0.90; 95% CI, 0.84-0.96) (Table 3). Lastly for assessing non–COVID-19 mortality in 2020, we further examined patient mortality prior to COVID-19 diagnosis, as a competing event of COVID-19 (eFigure 1 and eTable in the Supplement).

**Table 3.** Risk Factors for All-Cause Mortality Among Medicare Patients Receiving Dialysis With a COVID-19 Diagnosis

**Characteristic**	**HR (95% CI)**
Age (centered), per 10 y	1.37 (1.35-1.39)^a^
Sex	
Women	1 [Reference]
Men	1.20 (1.16-1.24)^a^
Race	
Black	0.87 (0.84-0.90)^a^
Non-Black^b^	1 [Reference]
Ethnicity	
Non-Hispanic	1 [Reference]
Hispanic	1.06 (1.02-1.10)^a^
BMI categories	
18.5-24.9	1 [Reference]
<18.5	1.11 (1.00-1.23)^a^
25.0-29.9	1.06 (1.01-1.11)^a^
≥30	1.05 (1.01-1.10)^a^
Locale	
Rural	1 [Reference]
Urban	0.90 (0.84-0.96)^a^
Nursing home stays in previous 365 d, d	
0s	1 [Reference]
1-89	1.31 (1.25-1.37)^a^
≥90	1.12 (1.07-1.16)^a^
Modality	
In-center hemodialysis	1 [Reference]
Home dialysis	1.18 (1.11-1.25)^a^
Medicare Advantage	1.30 (1.25-1.35)^a^
ESKD vintage (centered), per 10 y	1.10 (1.06-1.14)^a^
Atherosclerotic heart disease	1.05 (1.00-1.10)^a^
Other cardiac disease	1.03 (0.99-1.07)
Congestive Heart Failure	1.09 (1.05-1.13)^a^
Inability to ambulate	0.96 (0.89-1.04)
Chronic obstructive pulmonary disease	1.05 (0.99-1.12)
Inability to transfer	0.99 (0.89-1.09)
Malignant neoplasm, cancer	1.02 (0.95-1.09)
Diabetes	0.96 (0.92-1.00)
Peripheral vascular disease	1.00 (0.95-1.06)
Cerebrovascular disease (CVA, TIA)	0.98 (0.93-1.03)
Tobacco use (current smoker)	1.16 (1.07-1.25)^a^
Alcohol dependence	0.90 (0.77-1.05)
Drug dependence	0.88 (0.74-1.05)
Prevalent comorbidities, per 1-unit increase	1.04 (1.04-1.05)^a^
<180 d of Medicare claims in 2019	0.81 (0.77, 0.86)^a^

Abbreviations: BMI, body mass index (calculated as weight in kilograms divided by height in meters squared); CVA, cerebrovascular accident; ESKD, end-stage kidney disease; HR, hazard ratio; TIA, transient ischemic attack.

^a^ Indicates statistical significance.

^b^ Race was dichotomized to focus on the experience of Black patients. The non-Black reference group included White, Asian, Native Hawaiian or other Pacific Islander, and American Indian or Alaska Native patients and those who reported other race.

## Discussion

This cohort study examined COVID-19 and mortality among Medicare patients undergoing regular maintenance dialysis in 2020 to assess the outcomes associated with the pandemic in this high-risk population. Leveraging a comprehensive national database, we found differences in COVID-19 by race, ethnicity, and nursing home residence, as well as older age, higher BMI, in-center treatment, and several comorbid conditions. These observations are consistent with studies in the general population, although COVID-19 rates among patients receiving dialysis are notably higher.^10,28,29,41^ We also observed that mortality patterns in 2020 deviated from historical trends both overall and with respect to race, sex, and area of residence. Recent work has quantified this excess mortality among patients with ESKD, adjusting for patient age and geographic variation by ESRD Network.^29^ Older age, male sex, Hispanic ethnicity, nursing home residence, and higher BMI were also associated with higher postdiagnosis mortality in our study.

Much of the literature on COVID-19 has used regionally available data or has focused on other comorbid conditions. For example, in a major hospital near Detroit, Black patients, patients in urban areas, and patients with diabetes were reported to have higher odds of COVID-19.^7^ These results were similar to ours and further corroborated in recent work.^8,9^ Explanations may include disparity in social determinants of health, such as structural differences in socioeconomic status, employment, access to health care, or built environment that limit access to healthy foods and other health promoting activities.^42,43,44,45,46,47,48,49^

Importantly, we identified nursing home residence to be a significant risk factor associated with COVID-19 and subsequent death. Previous studies reported that nursing home residents, comprising less than 5% of the population, accounted for about 25% of COVID-19–related deaths nationwide.^50,51,52,53,54,55^ We estimated a 36% COVID-19 rate and 12% higher mortality among patients with extended stays vs patients with no prior time in a long-term care or skilled nursing facility. The nursing home experience during the pandemic appears to be particularly salient among the dialysis population, potentially owing to congregate living, the inability to socially distance, and higher frailty and comorbidity burden among nursing home residents.

Several recent studies provide evidence for worsened outcomes among patients with chronic kidney failure associated with COVID-19.^16,17,18,19,20,21,56,57,58^ Two independent international studies estimated a COVID-19 rate of 6% among patients undergoing dialysis and a 20% mortality rate.^59,60^ We estimated a 12% COVID-19 rate among US Medicare patients undergoing dialysis but, similarly, a 26% mortality rate after COVID-19. Few studies have examined risk factors for COVID-19 outcomes among patients undergoing dialysis. One study reported that patients with COVID-19 from a regional dialysis organization in New York were more likely to be older, male, Black, or Hispanic and to have longer times receiving dialysis.^61^ Another found that mortality among Hispanic patients with ESKD was 4-fold that of non-Hispanic patients.^62^ A study by Hsu et al^63^ based on patients from a midsize dialysis organization reported that male sex, Black race, treatment at an urban clinic, residence in a nursing home, and greater comorbidity burden were associated with COVID-19. Our results support these studies from the perspective of a national and more diverse chronic dialysis population. Lastly, recent national studies on hospitalization and mortality rates among patients with ESKD in the first wave of the pandemic similarly found significant differences in adjusted relative rates with respect to patient age, sex, race, and ethnicity.^64,65^

We further highlight several differences in risk factors between COVID-19 and mortality. First, higher BMI was associated with higher hazards for COVID-19 and mortality, consistent with findings in the general population.^2,4,7^ However, this contrasts the BMI-survival paradox we observe before COVID-19 diagnosis, which is well-studied in the literature.^66,67^ Second, while no significant differences were found in COVID-19 rates by sex, men had a 20% higher hazard for mortality once diagnosed. These results are similar in nondialysis populations.^68,69^ Patients undergoing dialysis at home had a 23% lower hazard for COVID-19, yet an 18% higher hazard of mortality after diagnosis. A recent study by Hsu et al^70^ reported on this difference in COVID-19 rates but suggested that the difference attenuated as COVID-19 has become more widespread. Hsu et al^70^ report a lower mortality rate among patients receiving dialysis at home; however, the hazards for mortality estimated in this study were comparable before vs after diagnosis.

Lastly, Black patients had a 25% higher hazard of COVID-19. It is possible that this difference may be owing to these patients disproportionately living in urban areas, residing in high-density housing, having frontline jobs, or possibly less access to health care.^7,46,47^ Black patients also had a 13% lower hazard of postdiagnosis mortality compared with non-Black patients. Out of context, this would suggest a protective association on survival. However, Black patients receiving dialysis have historically had much higher survival rates than non-Black patients prior to 2020^71,72,73^ and had an estimated 21% lower hazard of mortality prior to COVID-19 in this study. The attenuated survival difference after COVID-19 may suggest that COVID-19 may be associated with modifying survival outcomes for these patients.^28,74^ Therefore, it is likely a combination of higher COVID-19 incidence rates and attenuated survival differences that explains the disparate increase in early mortality rates observed among Black patients and the narrowing gap in mortality between Black and non-Black patients in 2020.

### Limitations

This study has several limitations. First, owing to delays in the processing of Medicare claims and lack of outpatient claims for Medicare Advantage patients, all events during the follow-up period may not yet be observed. We chose an administrative censoring date of December 31, 2020, four months prior to data extraction, to maximize the completeness of the data. While a longer follow-up is necessary to ascertain more information, these data are not yet available. We also note that these data would not include individuals with asymptomatic SARS-CoV-2 infection.^75^ Second, as incubation periods and onset of symptoms may differ, defining an exact infection date poses challenges. However, with no evidence of systematic biases, such imprecision is unlikely to change the results. Third, capturing COVID-19 cases is challenging, particularly before standardized conventions were adopted. To mitigate potential underreporting, we used string matching in physician write-ins to capture COVID-19 events. We also restricted our analysis to Medicare-eligible patients receiving dialysis. Patients undergoing long-term dialysis make up 70% of the total ESKD population, and Medicare-eligible patients make up 80% of the chronic dialysis population. Generalizability beyond our study population to the broader ESKD population would require additional data on non-Medicare patients and patients with ESKD who have received transplants. Additionally, states had varying testing and quarantine guidelines at different time points. Thus, there may be regional differences beyond the results we present nationally.

## Conclusions

To our knowledge, this cohort study is the first national study using CMS claims data to evaluate COVID-19 outcomes in the Medicare dialysis population using all available 2020 data through December 2020. Our results identified several risk factors for COVID-19 and mortality, which include nursing home residence, race, sex, modality, and several comorbidity conditions, such as diabetes and obesity. These results improve our understanding of COVID-19 and complications in this high-risk population and could inform policy decisions to mitigate the added burden of COVID-19 and death.

## Notes

Supplement. **eAppendix.** Data Extraction and Processing **eTable.** Risk Factors for All-Cause Mortality Prior to COVID-19 Diagnosis Among Medicare-Eligible Patients With ESKD Receiving Long-term Dialysis in 2020 **eFigure 1.** Diagram of the Relationship Among the 3 Models in the Analytic Workflow **eFigure 2.** Patient Subpopulations and Outcomes in the Study Sample
