# Error in Figure 1

**DOI:** 10.1001/jamanetworkopen.2022.19305

**Published:** 2022-06-08

In the Original Investigation titled “COVID-19 Risk Factors and Mortality Outcomes Among Medicare Patients Receiving Long-term Dialysis,”^1^ published November 17, 2021, the figure keys for Figure 1 were incorrect. This article has been corrected.^1^

## References

[1] Salerno S, Messana JM, Gremel GW, . COVID-19 risk factors and mortality outcomes among Medicare patients receiving long-term dialysis. JAMA Netw Open. 2021;4(11):e2135379. doi:10.1001/jamanetworkopen.2021.35379 34787655 PMC8600389

