# Supplementary material for: COVID-19 Risk Factors and Mortality Outcomes Among Medicare Patients Receiving Long-term Dialysis
Source: JAMA Netw Open. 2021 Nov 17;4(11):e2135379. doi: 10.1001/jamanetworkopen.2021.35379 (PMC8600389; doi:10.1001/jamanetworkopen.2021.35379)
Supplement: Supplement. — eAppendix. Data Extraction and Processing eTable. Risk Factors for All-Cause Mortality Prior to COVID-19 Diagnosis Among Medicare-Eligible Patients With ESKD Receiving Long-term Dialysis in 2020 eFigure 1. Diagram of the Relationship Among the 3 Models in the Analytic Workflow eFigure 2. Patient Subpopulations and Outcomes in the Study Sample [file jamanetwopen-e2135379-s001.pdf]

Supplemental Online Content

Salerno S, Messana JM, Gremel GW, et al. COVID-19 risk factors and mortality outcomes among Medicare patients receiving long-term dialysis. *JAMA Netw Open*. 2021;4(11):e2135379. doi:10.1001/jamanetworkopen.2021.35379

eAppendix. Data Extraction and Processing

eTable. Risk Factors for All-Cause Mortality Prior to COVID-19 Diagnosis Among Medicare-Eligible Patients With ESKD Receiving Long-term Dialysis in 2020

eFigure 1. Diagram of the Relationship Among the 3 Models in the Analytic Workflow

eFigure 2. Patient Subpopulations and Outcomes in the Study Sample

This supplemental material has been provided by the authors to give readers additional information about their work.

© 2021 Salerno S et al. *JAMA Network Open.*

eAppendix. Data Extraction and Processing

A.1.1 Identification of Potential Risk Factors

The set of risk factors utilized in all three Cox models were derived in alignment with the methodology for calculating the Standardized Mortality Ratio for Dialysis Facilities (SMR), as reported on Care Compare: https://www.medicare.gov/care-compare/. A detailed description of the SMR methodology, including data sources for the covariates used in this study, can be found at: https://dialysisdata.org/sites/default/files/content/ESRD_Measures/SMR2016.pdf. The SMR undergoes rigorous measure testing and maintenance for endorsement by the National Quality Forum: https://www.qualityforum.org/home.aspx.

A.1.2 Choice of Identified Prevalent Comorbidities

Consideration of prevalent comorbidities as risk adjusters, in addition to incident comorbidities, is in part a response to stakeholder interest to adjust for more current (prevalent) comorbidities to reflect the current health status of dialysis patients, and conditions associated with mortality. A Technical Expert Panel (TEP) was convened to consider which addition of prevalent comorbidities should be included in public-facing risk-adjusted mortality models. The summary report for the TEP can be found here: https://dialysisdata.org/content/esrd-measures. This process resulted in the TEP recommending a list of 210 individual ICD-9 diagnosis codes for inclusion as risk adjustors. With the expansion of diagnostic codes that accompanied the transition from ICD-9 to ICD-10 in 2015, the original list of 210 comorbidities grew to over 1,000 ICD-10 codes. We collapsed the original 210 individual ICD-9 codes into 90 clinical groups using the Agency for Healthcare Research and Quality (AHRQ) Clinical Classifications Software (CCS) categories as the framework for grouping the selected prevalent comorbidities. Using a crosswalk, the ICD-10 codes were then mapped to the 90 clinical comorbidity groups that are included in the prevalent comorbidity score (number of prevalent comorbidities) for our analytic models. Further details can be found at: https://dialysisdata.org/sites/default/files/content/ESRD_Measures/SMR2016.pdf

A2. Additional Analysis Results

To fully capture a patient's potential transitions to COVID-19 morbidity and mortality (see Supplementary Figure S1), we fit a third model which examined the competing risk of patient

© 2021 Salerno S et al. *JAMA Network Open.*

mortality prior to COVID-19 infection. Here, we modeled time to death, which could be censored by infection, kidney transplantation, Medicare ineligibility, or the end of follow up. We report the adjusted hazard ratios (HR) and 95% confidence intervals (95% CI) from this Model 3 for the risk factors defined previously in Table S1 below.

© 2021 Salerno S et al. *JAMA Network Open.*

eTable. Risk Factors for All-Cause Mortality Prior to COVID-19 Diagnosis Among Medicare-Eligible Patients With ESKD Receiving Long-term Dialysis in 2020

Time at risk was taken from January 1, 2020, or start of dialysis, whichever came later, until death, which could be censored by COVID-19 infection, kidney transplantation, loss of Medicare eligibility, or administrative censoring on December 31, 2020.

Characteristic	HR (95% CI)[1]
Age (centered, per 10 years)	**1.34 (1.33-1.34)** [2]
Sex	
Female	Ref.[1]
Male	**1.10 (1.08-1.11)**
Race	
Non-Black	Ref.
Black	**0.79 (0.78-0.81)**
Ethnicity	
Non-Hispanic	Ref.
Hispanic	**0.83 (0.82-0.85)**
Body Mass Index Categories	
Normal	Ref.
Underweight	**1.17 (1.12-1.23)**
Overweight	**0.93 (0.91-0.95)**
Obese	**0.89 (0.88-0.91)**
Nursing Home Stay in Previous 365 Days	
0 Days	Ref.
1-89 Days	**1.57 (1.54-1.61)**
90+ Days	**1.72 (1.68, 1.77)**
Urban versus Rural Locale	**0.88 (0.85-0.90)**
Home Dialysis versus In-Center Hemodialysis	**1.19 (1.17-1.22)**
Medicare Advantage	**0.98 (0.96-0.99)**
ESRD Vintage (centered, per 10 years)	**1.02 (1.00-1.04)**
Atherosclerotic Heart Disease	**1.07 (1.04-1.09)**
Other Cardiac Disease	**1.06 (1.04-1.08)**
Congestive Heart Failure	**1.11 (1.09-1.13)**
Inability to Ambulate	**1.08 (1.04-1.12)**
Chronic Obstructive Pulmonary Disease	**1.05 (1.02-1.08)**
Inability to Transfer	**1.07 (1.01-1.13)**
Malignant Neoplasm, Cancer	**1.11 (1.08-1.14)**
Diabetes (Cause of ESRD)	**0.97 (0.95-0.99)**
Peripheral Vascular Disease	**1.10 (1.07-1.13)**
Cerebrovascular Disease (CVA, TIA)	1.00 (0.98-1.03)
Tobacco Use (Current Smoker)	**1.22 (1.18-1.26)**
Alcohol Dependence	1.05 (0.98-1.12)
Drug Dependence	**1.15 (1.06-1.24)**
Number of Prevalent Comorbidities	**1.13 (1.13-1.13)**
Less Than 180 Days of Medicare Claims in 2019	**1.10 (1.07-1.12)**

[1]HR = Hazard Ratio; CI = Confidence Interval; Ref. = Reference Group
[2]Bold values indicate statistical significance

eFigure 1. Diagram of the Relationship Among the 3 Models in the Analytic Workflow

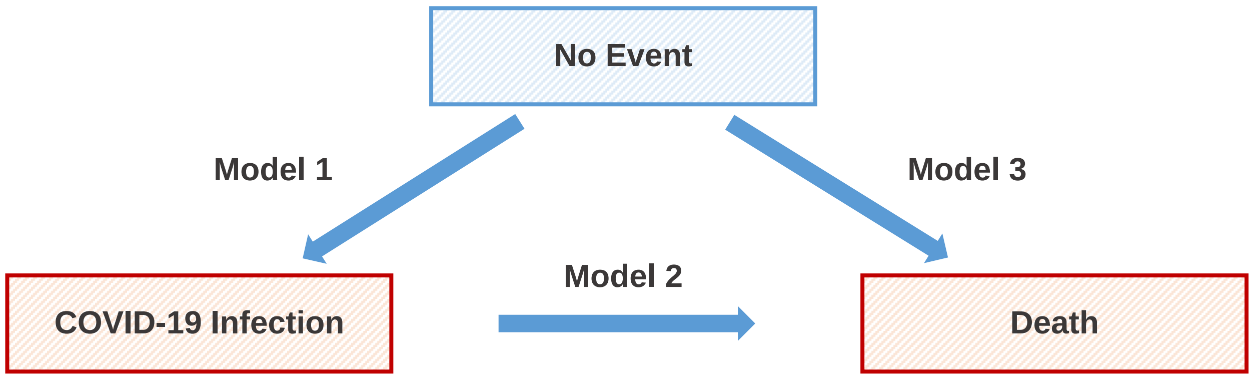

eFigure 2. Patient Subpopulations and Outcomes in the Study Sample

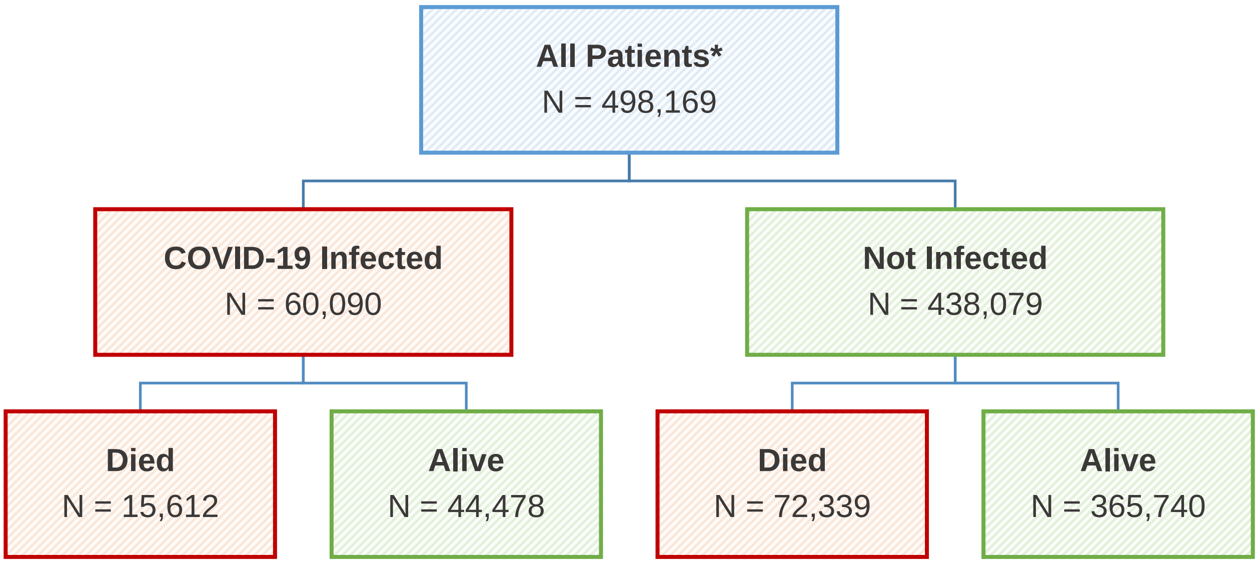

*Medicare-eligible patients with time at risk on chronic dialysis between Jan. 1 and Dec. 31, 2020

© 2021 Salerno S et al. *JAMA Network Open.*
